# Development of SmokeFree Baby: a smoking cessation smartphone app for pregnant smokers

**DOI:** 10.1007/s13142-016-0438-0

**Published:** 2016-10-03

**Authors:** Ildiko Tombor, Lion Shahab, Jamie Brown, David Crane, Susan Michie, Robert West

**Affiliations:** 1 Cancer Research UK Health Behaviour Research Centre, University College London, WC1E 6BT, London, UK; 2 Department of Epidemiology and Public Health, University College London, WC1E 6BT, London, UK; 3 Department of Clinical, Educational and Health Psychology, University College London, WC1E 6BT, London, UK

**Keywords:** Digital interventions, Smartphone application, Behaviour change interventions, Smoking cessation, Pregnant smokers, Incremental development

## Abstract

Pregnant smokers may benefit from digital smoking cessation interventions, but few have been designed for this population. The aim was to transparently report the development of a smartphone app designed to aid smoking cessation during pregnancy. The development of a smartphone app (‘SmokeFree Baby’) to help pregnant women stop smoking was guided by frameworks for developing complex interventions, including the Medical Research Council (MRC), Multiphase Optimization Strategy (MOST) and Behaviour Change Wheel (BCW). Two integrative behaviour change theories provided the theoretical base. Evidence from the scientific literature and behaviour change techniques (BCTs) from the BCT Taxonomy v1 informed the intervention content. The app was developed around five core modules, each with a distinct intervention target (identity change, stress management, health information, promoting use of face-to-face support and behavioural substitution) and available in a ‘control’ or ‘full’ version. SmokeFree Baby has been developed as part of a multiphase intervention optimization to identify the optimum combination of intervention components to include in smartphone apps to help pregnant smokers stop smoking.

**Electronic supplementary material:**

The online version of this article (doi:10.1007/s13142-016-0438-0) contains supplementary material, which is available to authorized users.

## Introduction

Maternal smoking is detrimental to foetal development and is the leading avoidable cause of prenatal and neonatal mortality and morbidity in high-income Western countries [1, 2]. Behavioural support [3] and a combination of different forms of nicotine replacement therapy (NRT) [4] are effective in helping pregnant women quit smoking, but challenges in access to effective care remain. For example, in the financial year of 2014–2015, only 18,887 pregnant smokers (approximately 26.6 % of pregnant smokers) set a quit date at face-to-face smoking cessation services in England [5, 6]. Potential barriers to engaging with face-to-face support involve fear of being judged, poor access to health care facilities and negative attitudes towards the support provided by health care providers [7–9]. It is important to find alternative ways of delivering smoking cessation support that may appeal to pregnant women. Digital interventions represent a viable option due to their wide reach and potential to afford users anonymity and convenience; however, there is little known about what intervention components are most likely to constitute an effective intervention. This paper describes the development of a pregnancy-specific smoking cessation app (‘SmokeFree Baby’) to provide a basis for intervention optimization.

Web sites and text-messaging programmes can be effective for increasing quit rates in the general adult population [10, 11], and recent pilot studies have reported promising results with theory-driven and evidence-based smartphone apps to aid cessation [12–14]. Only a few digital smoking cessation interventions have been designed specifically for pregnant smokers, but both Web sites and text-messaging programmes [15–18] have been found to be acceptable and potentially engaging in this population, and pilot comparative trials have shown positive but not statistically significant effects of these interventions [19–21]. However, to the best of our knowledge, no study has been published on the development or evaluation of smoking cessation apps for pregnant smokers.

Multiphase approaches to developing and evaluating behaviour change interventions, such as the UK’s Medical Research Council (MRC) guidance [22], the Multiphase Optimization Strategy (MOST) [23] and the Behaviour Change Wheel (BCW) [24], suggest that multicomponent interventions should be developed systematically through a number of iterative phases of intervention component selection, pilot evaluations and randomized controlled trials (RCTs), with the implementation phase feeding back to a new cycle of intervention development. A key tenet of all the aforementioned methodological frameworks is to identify potential intervention components by a systematic application of theory and scientific evidence [22–24]. Theoretical frameworks can be used to explain the mechanisms through which the intervention is expected to influence behaviour change, and digital interventions embedded in theory have been found to be more effective compared with interventions that used theory less extensively [25]. The BCW [24] recommends assessing both the target behaviour and ways in which people and/or their environment need to change to alter that behaviour so that the content of the intervention can be specified accordingly. MOST [23] suggests that the effects of selected intervention components need to be tested experimentally in order to inform decisions regarding an optimal set of components and component levels that would be expected to constitute an effective intervention.

The development of a smoking cessation smartphone app reported in this paper followed the above principles, and it involved the following steps: (1) identifying the target behaviours, (2) identifying the theoretical base, (3) reviewing relevant scientific literature, (4) conducting exploratory work and need assessment, (5) selecting mode of delivery, (6) selecting intervention components, (7) specifying the intervention content by BCTs, (8) translating the intervention content into app features, (9) designing a prototype intervention and (10) piloting the app before its launch.

Transparency in reporting the development and content of complex interventions is paramount for understanding intervention effects and accumulating evidence in the field of digital intervention science [26]. This paper provides a comprehensive description of (i) the intervention development of a smoking cessation smartphone app for pregnant smokers and (ii) the ways in which methodological and theoretical frameworks and evidence from the scientific literature have been translated into specific intervention components in the app.

## Methods

The intervention development was informed by the MRC guidance, MOST and BCW overarching methodological frameworks [22–24]. Figure S1 in the supplementary files shows the process of intervention development according to a multiphase approach. It comprised three main phases, which were further divided into a total of ten steps as follows: preparation phase (steps 1–4), design phase (steps 5–8) and piloting phase (step 10). Details of each step are reported below.

### Step 1. Identify target behaviours

Complete cessation during pregnancy was selected as the primary target behaviour of the intervention, because it yields the greatest health benefits both for pregnant women and their children [27, 28]. For those who cannot or do not want to stop in one step, a secondary target behaviour of reducing smoking to three or fewer cigarettes per day was also included for the following reasons. First, a dose-response relationship has been found between the overall prenatal tobacco exposure and infant birth weight [29], and it has been suggested that a substantial smoking reduction (e.g. cutting down to 2–3 cigarettes per day) can be associated with increased birth weight [28, 30, 31]. Secondly, by recognizing that pregnant smokers have low self-confidence in their ability to stop smoking [32, 33], a smoking reduction option may give pregnant smokers the opportunity to gain confidence and increase self-efficacy before trying to quit completely. Thirdly, smoking cessation can involve multiple attempts to stop smoking and to try to cut down [34], and pregnant smokers may make a number of quit attempts during pregnancy [35]; therefore, it is important to engage them with cessation support even if they lapse or relapse. Lastly, a meta-analysis showed that smokers who want to quit smoking and cut down prior to complete cessation are as likely to be abstinent at 6 months as those who quit abruptly [36]. In addition, a population survey found that in the general population of smokers, those who reduce smoking have higher odds of quit attempts and cessation at 6 months than those who do not cut down [37]. Using NRT for smoking, reduction can also promote cessation [37].

### Step 2. Identify the theoretical base

We drew on two integrative behaviour change theories that provide comprehensive frameworks to understand behaviour and behavioural patterns in context, as well as the influences that can bring about change in behaviours. First, the Capability, Opportunity, Motivation and Behaviour (COM-B) model [38] suggests that at any moment, three interacting conditions are necessary for any behaviour to occur: people need to have the necessary ‘capability’ (psychological and physical capability) to perform the behaviour, ‘opportunity’ (afforded by the social and physical environment) to engage in the behaviour and strong enough ‘motivation’ (automatic and reflective) to generate the behaviour.

The second was a broad motivational theory: PRIME which stands for ‘Plans’, ‘Responses’, ‘Impulses/Inhibitions’, ‘Motives’ and ‘Evaluations’. A hierarchically structured human motivational system is proposed, in which ‘responses’ (e.g. smoking a cigarette) are on the lowest level, then ‘impulses/inhibitions’ (e.g. an urge to smoke in the presence of smoking cues), ‘motives’ (e.g. the want or need to smoke), ‘evaluations’ (e.g. a belief that smoking eliminates stress; or thinking about oneself as a non-smoker) and ‘plans’ (e.g. a plan to stop smoking). The system as a whole operates on a moment-to-moment basis and higher levels of the motivational system can only energize and direct behaviour by influencing lower levels.

Key tenets of the COM-B model [38] and PRIME theory [39] were applied to inform intervention component selection; the principles generated from these theories are reported in the supplementary tables (Table S1). For example, COM-B suggests that knowledge can be an important factor in bringing about change in behaviour; therefore, providing information about different types of smoking cessation support was identified as a potential intervention target. PRIME theory argues that maintaining desired behaviour change requires mental energy and for the individual to exercise self-control in situations when competing wants and needs to smoke arise. One way to conserve mental energy and cope with momentary desires to smoke is by engaging with an alternative behaviour, and it was identified that the intervention needs to provide distraction from urges to smoke and improve pregnant smokers’ skills to substitute smoking with alternative behaviours. PRIME theory recognizes identity as an important source of motivation; thus, fostering a non-smoker identity was also selected as a core component of the intervention.

### Step 3. Review relevant scientific literature

In addition to theoretical principles, intervention components were selected based on evidence from the fields of smoking cessation and behaviour change research. This included behaviour change techniques (BCTs) that were previously identified in treatment manuals of behavioural support provided by the English Stop Smoking Services, a nationwide network of specialist services providing behavioural support and pharmacotherapy to aid cessation, and were found to be associated with short-term quit success (e.g. strengthen ex-smoker identity) [40]. Additionally, BCTs that were identified in intervention descriptions of effective behavioural support for pregnant smokers (e.g. self-monitoring of behaviour) were included [41]. Systematic literature searches were conducted via PubMed in relation to smoking in pregnancy, interventions for pregnant smokers and digital cessation aids. This identified, for example, that stress management [42–44], information about the health consequences of smoking [45–47] and tips to avoid social cues for smoking [48] might be important to include. Although providing financial incentives can also be effective among pregnant smokers [3, 44, 49–51], it was not feasible to include financial incentives in an app offering automated support.

### Step 4. Conduct exploratory work and need assessment

The exploratory work involved (1) focus groups with health care providers (HCPs) [52] to solicit their views on how digital interventions should be configured in order to improve pregnant smokers’ cessation efforts and (2) interviews with pregnant smokers [53] using the COM-B framework [38] to identify what would need to change in pregnant smokers and/or their environment in order for them to stop smoking. HCPs, including stop smoking advisors and midwives who provide smoking cessation support for pregnant smokers, were recruited because of their potentially valuable insights into effective methods to aid cessation during pregnancy. For example, HCPs recommended that the intervention should increase pregnant smokers’ motivation and confidence to quit by establishing rewarding experiences (e.g. providing badges as rewards) [52], and pregnant smokers emphasized that having easy access to further cessation support (preferably face-to-face) would be important [53].

### Step 5. Select mode of delivery

The BCW recommends selecting the mode of delivery to promote the intervention being delivered affordably, practicably, cost-effectively, acceptably, safely and equitably across the target population [24]. We chose to develop a smartphone app (SmokeFree Baby) for the following reasons: (1) the intervention could be delivered on a relatively low cost per user and low marginal cost; if demonstrated to be effective, a fully automated smoking cessation intervention could be highly cost-effective; (2) it could provide ready access to cessation support; (3) it could reach pregnant smokers who might otherwise be missed, since they do not engage with face-to-face support; 4) it could permit increased fidelity in intervention delivery; and (5) apps represent more advanced technology than Web sites or text-messaging, as they can take full advantages of a multi-touch interface and other functionalities of smart digital devices. SmokeFree Baby will be available from app stores for free and operational on Android 4.1 or later and iOS 6.0 or later for both smartphones and tablets.

### Step 6. Select intervention components

A set of intervention components was selected, informed by steps 1–4 in the preparation phase and expert consensus in the research team. Some were general app features aimed at all users, and some were core modules to which users are randomly allocated. A ‘module’ refers to the unit of intervention components to be tested experimentally in the app. For each module, the content of a control and full version was also specified. Developing a control and full version of each module was to ensure that all participants receive all intervention content to some extent. A control version was intended to provide minimal credible control against which to compare the full version of the module. For all general app features and core modules, key targets (e.g. prompt participants to record how many cigarettes they smoke each day and provide distraction) were specified. The intervention functions likely to be effective for these targets were identified from the BCW (e.g. persuasion and enablement) [24].

### Step 7. Specify the intervention content by BCTs

The BCT Taxonomy v1 (BCTTv1) [54] was applied to select BCTs judged to be suitable to deliver the content in the app given the key targets and intervention functions. The BCTTv1 is a comprehensive taxonomy, which has been systematically developed based on expert consensus. It provides a cross-domain and hierarchically structured list of 93 distinct BCTs, each with a label, definition and example.

### Step 8. Translate the intervention content into app features

Translating the intervention content into app features involved repeated discussions between members of the research team and the app development team. Several iterations of SmokeFree Baby were produced until agreement was reached that both the general app features and core modules could deliver the intervention content as intended, and the features were feasible to implement in terms of computer programming.

### Step 9. Design a prototype intervention

To promote user engagement, design strategies relating to persuasion (e.g. aesthetics, reminders) and usability (e.g. easy navigation) were applied [55]. Principles that informed the design of the app reported the supplementary tables (Table S2). Twenty-three principles were adopted from the development of the ‘StopAdvisor’ smoking cessation Web site [56] (e.g. the text for the app was edited by a professional writer to ensure that it was as brief as possible and easy to understand). Three principles were adopted from a study exploring optimal features of health-related Web sites [57] (e.g. keep the background questionnaire short and present it with a progress bar in order to minimize respondent burden and avoid early dropouts). Two principles were identified in a study soliciting HCPs’ recommendations regarding digital aids in pregnancy [52] (e.g. provide daily tips). The research team identified further eight principles, such as using push notifications, placing greater emphasis on the full modules by including visuals and interactive elements and using text only in the control versions. The app development team was provided with an intervention specification document detailing the content, design and operation of specific app features.

### Step 10. Pilot the app

The content of the app was checked against the intervention specification by IT. In order to test all features in an iterative manner and identify and fix programming bugs, an initial user testing was also conducted within the research team and with a convenience sample of non-pregnant users (*n* = 6). Everyone was given access to a test version of the app and was asked to email comments and feedback on the app to IT. Following discussions within the research team, the app was refined before its launch. A number of modifications were made to correct errors (e.g. correct feedback on progress), amend the design (e.g. increase font size), improve user experience (e.g. improve navigation within built-in features) and ensure the stability of the app on different iOS and Android software versions.

## Results

The SmokeFree Baby app has been designed to help pregnant smokers stop smoking or cut down. It provides automated support throughout pregnancy without face-to-face contact and includes general app features and five core modules (each in a control and full version). Each module has a specific topic, and within each module, various BCTs are used to deliver the intervention content accordingly. Sample screenshots of the app are reported in the supplementary files (Fig. S2), and details of the content are discussed below.

### Registration and general app features

**Fig. 1:**
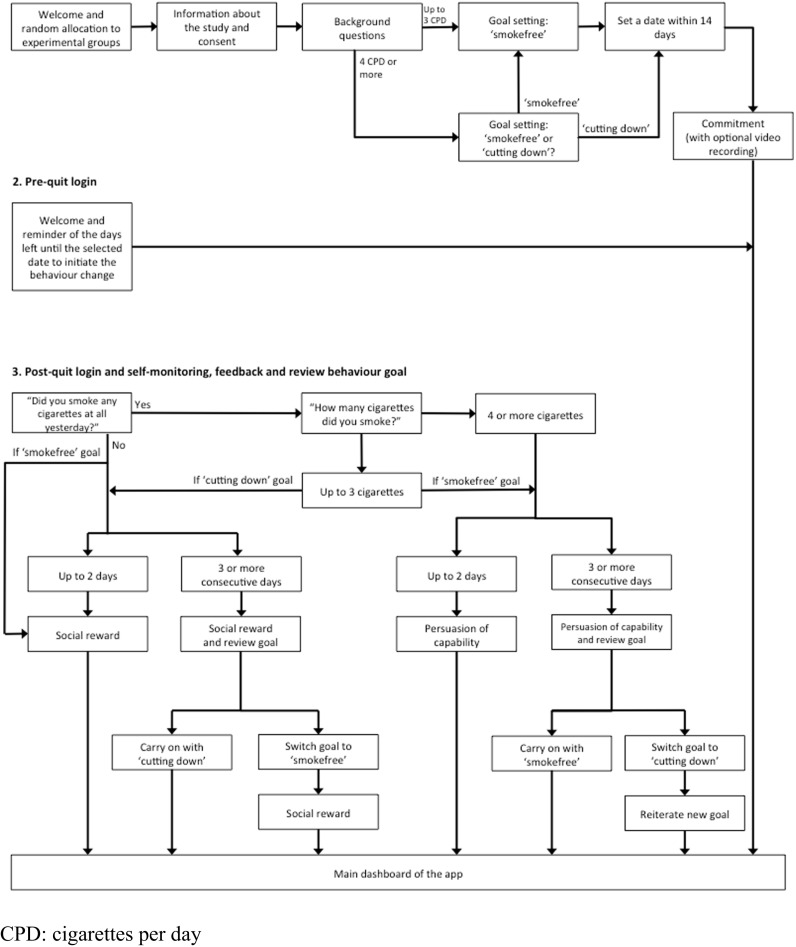
Registration and subsequent logins. *CPD* cigarettes per day

Figure 1 shows the process of registration and subsequent logins to the app. Pregnant women are asked to read information about the study and, if they consent to participate, complete a baseline questionnaire. Participants who smoke four or more cigarettes per day at baseline can decide if they want to stop smoking completely or cut down to three or fewer cigarettes per day. Those who smoke three or fewer cigarettes can only select the ‘smokefree’ goal. Everyone is advised to set a date to initiate behaviour change within 2 weeks of completing the registration and affirm commitment to that goal. Participants can use the app preceding their chosen date, and they are reminded of the days remaining before that date. Once the day of initiating behaviour change has past, the first login each day starts with asking participants if they smoked any cigarettes at all in the last 24 h, and if so, how many. Depending on their response, participants are praised for their success or given supportive messages. Those who cut down successfully for three consecutive days are encouraged to try to stop smoking completely, and those who do not manage to stay abstinent for three consecutive days are offered to change their target behaviour to smoking reduction.

The general app features are summarized in Table 1. Features that are available for all participants before initiating behaviour change aim to (1) provide information about the app, the research team, nicotine addiction and withdrawal symptoms and (2) prompt participants to think about why they want to quit and how to prepare for changing their behaviour. Other general features include a saving calculator to monitor how much money they have saved since quitting or cutting down, advice on using NRT and advice to engage in social situations without smoking.

**Table 1:** Key targets in general app features with proposed process of change, intervention functions and behaviour change techniques (BCTs)

Features	Key targets	Proposed process of change ^a^	Intervention functions ^b^	BCTs ^c^	Sample text
Goal setting	• Set a behaviour change goal: stopping smoking or cutting down • Prompt participants to affirm commitment to their selected goal • Review goal and advise participants to consider modifying it if needed	• Psychological capability: self-regulation • Reflective motivation: intentions; goals	Enablement Persuasion	1.1 Goal setting (behaviour) 1.5 Review behaviour goal 1.6 Discrepancy between current behaviour and goal 1.9 Commitment	“As you are progressing very well here, it’s a good time to revisit your initial goal and think about stopping smoking completely. You can do this!”
Feedback and monitoring	• Prompt participants to record how many cigarettes they smoke each day • Provide feedback on performance of the selected behaviour • Praise participants for progress in changing the behaviour • Prompt self-praise and self-reward • Boost motivation and self-confidence • Advise participants to think about previous successes with quitting	• Psychological capability: self-regulation • Environmental opportunity: resources • Automatic motivation: habit formation; reinforcement • Reflective motivation: self-confidence	Enablement Persuasion	2.2 Feedback on behaviour 2.3 Self-monitoring of behaviour 10.4 Social reward 10.7 Self-incentive 10.9 Self-reward 15.1 Verbal persuasion about capability 15.3 Focus on past success	“You have reached your one-week milestone. Sounds like a great achievement! Why do not you think about a reward for yourself if you stick to your smokefree goal for the next 7 days?”
Features available pre-quit	• Provide information about the app, the research team, nicotine addiction and withdrawal symptoms • Prompt participants to think about why they want to quit smoking • Prompt planning of performing the behaviour	• Psychological capability: knowledge; planning • Automatic motivation: desires	Education Persuasion	1.4 Action planning 3.1 Social support (unspecified) 5.3 Information about the social and environmental consequences 11.1 Pharmacological support 12.1 Restructuring the physical environment 12.2 Restructuring the social environment 12.3 Avoidance/reducing exposure to cues for the behaviour	“SmokeFree Baby has been developed by a research team at University College London who specialize in smoking cessation.”
Savings calculator	• Monitor and provide feedback on how much money participants have saved	• Automatic motivation: reinforcement	Persuasion	2.7 Feedback on outcome of behaviour	“Saved so far up to £10. That’s enough to buy a baby bottle.”
‘Medicine’	• Provide information about smoking cessation medications	• Psychological capability: knowledge	Education	11.1 Pharmacological support	“It is best to combine mouth spray with […] nicotine patches.”
‘Support’	• Advise on eliciting social support	• Social opportunity: social influence	Enablement	1.4 Action planning 3.1 Social support (unspecified)	“Think about the people closest to you who you can rely on when you need support. Add their phone numbers here and call them if you feel that the urge to smoke is getting overwhelming.”
‘Memos’	• Advise on eliciting social support • Prompt participants to reaffirm their commitment with the behaviour change	• Social opportunity: social influence • Reflective motivation: intentions; goals	Enablement Persuasion	3.1 Social support (unspecified) 1.9 Commitment	“Help maintain your motivation to stop smoking or cut down by recording supportive video messages from your friends and family. You can also record your personal commitment to the goal you set for yourself.”
‘Social’	• Provide information about cues and social situations that can trigger urges to smoke • Provide tips to avoid environmental and social cues of smoking • Advise on eliciting social support	• Psychological capability: knowledge; self-regulation; planning • Social opportunity: social influence; social cues • Environmental opportunity: environmental cues • Reflective motivation: beliefs about consequences	Education Persuasion Environmental restructuring	1.4 Action planning 3.1 Social support (unspecified) 3.2 Social support (practical) 3.3 Social support (emotional) 4.2 Information about antecedents 5.3 Information about the social and environmental consequences 6.3 Information about others’ approval 12.1 Restructuring the physical environment 12.2 Restructuring the social environment 12.3 Avoidance/reducing exposure to cues for the behaviour 15.2 Mental rehearsal of successful performance 16.2. Imaginary reward 16.3. Vicarious consequences	“Imagine that you are out with friends and you are the only one who does not smoke. Prepare in advance what you are going to do when they go to have a cigarette. For example you can browse the internet on your phone to kill time.”

^a^based on the COM-B model [38] and PRIME theory [39]; ^b^based on the BCW [24]; ^c^selected from the BCTTv1 [54]

### Core intervention modules

Table 2 reports the specification of the core intervention modules. The structure of the modules is reported in the supplementary files (Fig. S3), and the content is discussed below.

1. ‘Identity’ module

**Table 2:** Core intervention modules, proposed process of change, intervention functions and behaviour change techniques (BCTs)

Modules	Key targets	Proposed process of change ^a^	Intervention functions ^b^	BCTs ^c^	Sample text
‘Identity’					
Control	• Foster identity change	• Reflective motivation: identity	Persuasion	13.5 Identity associated with changed behaviour	“Building up a new identity as someone for whom smoking is not an option any more is an important part of leaving smoking behind for good.”
Full	• Foster identity change • Prompt positive self-labels, self-images and self-thoughts • Increase salience of identities that do not promote smoking • Prompt identification with positive role models for cessation • Facilitate bonding with the baby	• Reflective motivation: identity; self-esteem; beliefs about consequences • Automatic motivation: desires • Social opportunity: social influence; modelling	Persuasion Modelling	13.5 Identity associated with changed behaviour 13.4 Valued self-identity 13.3 Incompatible beliefs 13.2 Framing/reframing 13.1 Identification of self as a role model 3.1 Social support (unspecified) 9.3 Comparative imagining of future outcomes 15.4 Self talk	“You might feel that smoking has always been a part of who you are, and stopping smoking would mean that you lose something of yourself. Think about what you can gain by making ‘not smoking’ an essential part of your identity. Make a list of all the things about yourself that will not change even if you become a non-smoker.”
‘Stress relief’					
Control	• Provide information about smoking and stress • Advise on using stress management techniques	• Psychological capability: knowledge; self-regulation	Education	11.2 Reduce negative emotions 12.6 Body changes	“Bear in mind that smoking does not reduce stress, it simply relieves your withdrawal symptoms.”
Full	• Provide information about smoking and stress • Advise on using stress management techniques • Provide training to perform a brief breathing exercise • Prompt planning to cope with stress without smoking	• Psychological capability: knowledge; self-regulation; skills; memory processes; planning • Reflective motivation: beliefs about consequences	Education Training Enablement	11.2 Reduce negative emotions 12.6 Body changes 1.2 Problem solving 1.4 Action planning 8.3 Habit formation 11.3 Conserving mental resources	“Here are 3 top tips to reduce your stress without smoking. Try them out the next time you begin to feel stressed.”
‘Health effects’					
Control	• Provide information about the health effects of smoking and benefits of cessation	• Psychological capability: knowledge • Automatic motivation: desires	Education Persuasion	5.1 Information about health consequences	“By stopping smoking completely during pregnancy […] your baby is less likely to be born too early with a low birth weight.”
Full	• Provide information about the health effects of smoking and benefits of cessation • Address misconceptions about the effects of smoking	• Psychological capability: knowledge • Automatic motivation: desires • Reflective motivation: beliefs about consequences	Education Persuasion	5.1 Information about health consequences 5.3 Information about social and environmental consequences 5.6 Information about emotional consequences	“Delivering a baby with a low birth weight is the main pregnancy complication known to be linked with both active and passive smoking. The less you smoke, the greater your chances of having a normal birth are.”
‘Face-to-face’					
Control	• Provide information about sources of support and how to access them	• Psychological capability: knowledge	Education	3.1 Social support (unspecified)	“One of the most effective ways of stopping smoking is by getting support from an expert stop smoking advisor.”
Full	• Provide information about sources of support and how to access them • Provide ready access to support in the localities • Address misconceptions about face-to-face support • Advise on making an appointment	• Psychological capability: knowledge • Environmental opportunity: access to support • Social opportunity: social influence	Education Persuasion Enablement	3.1 Social support (unspecified) 3.2 Social support (practical)	“It’s never too late to change your mind about getting face-to-face support from a stop smoking advisor. Even if you decided not to get support at first, you can always ask your GP or midwife for a referral to your local stop smoking clinic, or contact them yourself.”
‘Behaviour’					
Control	• Provide information about sources of urges to smoke • Promote behavioural substitution	• Psychological capability: knowledge; self-regulation	Education	4.2 Information about antecedents 8.2 Behaviour substitution 12.4 Distraction	“Have a think about what you can do instead of smoking. One option might be to play a game on your phone.”
Full	• Provide information about sources of urges to smoke • Promote behavioural substitution • Provide distraction • Prompt planning to cope with urges to smoke	• Psychological capability: knowledge; self-regulation; planning; memory processes • Environmental opportunity: resources • Automatic motivation: habit formation	Education Training Enablement	4.2 Information about antecedents 8.2 Behaviour substitution 12.4 Distraction 1.2 Problem solving 1.4 Action planning 8.3 Habit formation	“Decorate your baby’s room. Search on the internet for ideas then try to do little bits and pieces every time you think about smoking”

^a^Based on the COM-B model [38] and PRIME theory [39]; ^b^based on the BCW [24]; ^c^selected from the BCTTv1 [54]

The control version contains brief advice on establishing a mental image of becoming a non-smoker. The full version provides further motivational messages to support identity change in smoking cessation and aims to increase the salience of a positive identity in relation to the behaviour change through interactive features. In order to prompt identification with positive role models for cessation, video clips with an ex-smoker pregnant woman are included who talks about her experiences, struggles and successes with stopping smoking. In order to increase the salience of a ‘mother’ identity and facilitate bonding with the baby, information about foetal development is provided each week (tailored to the individual’s stage of pregnancy), and participants are encouraged to document their pregnancy using a video diary feature.

2. ‘Stress relief’ module

The control version provides brief information about the association between smoking and stress, and participants are given advice on using stress management techniques to cope with cravings. The full version adds to these by promoting the development of plans to prepare for coping with stressful situations and negative emotional states. A variety of tips are also provided that participants could use to create their own stress management plan within the app. In order to improve stress management skills, an interactive feature is included to train participants to perform a deep breathing exercise.

3. ‘Health effects’ module

Brief information about the health effects of smoking during pregnancy and the benefits of cessation is included in the control version. The full version, in which the content is delivered through quizzes, daily ‘health facts’ and interactive visuals that allow participants to explore as much or as little as they want of the available information, provides a comprehensive overview of the harmful effects of active and passive smoking both for mothers and their children. In order to minimize potential emotional distress for pregnant women, the messages are framed around the potential short-term and long-term benefits that could be gained by quitting.

4. ‘Face-to-face’ module

In the control version, participants are given brief information about evidence-based face-to-face support for cessation and how they could book an appointment with an expert stop smoking advisor. The full version provides further advice to encourage participants to engage with face-to-face support and includes video clips of a real-life specialist ‘stop smoking in pregnancy’ advisor who explains what face-to-face support involves, what pregnant smokers can expect when they make an appointment and how expert advisors can help them. Easy access to quitlines and local services in the UK, USA, Canada, Australia, New Zealand and the Republic of Ireland are also provided through the full version.

5. Behavioural substitution

The control version provides brief information about internal and external sources of urges to smoke and promotes behavioural substitution by means of using distraction strategies to cope with urges. In the full version, participants are encouraged to create their own distraction plan by using the tips provided. In order to help them distract their attention from urges, quizzes and a built-in game are included.

## Discussion

The SmokeFree Baby app has been designed to target a broad range of influences on behaviour, including psychological capability (e.g. improve self-regulation), social opportunity (e.g. provide role models for cessation), environmental opportunity (e.g. provide ready access to cessation support), automatic motivation (e.g. increase desires to stop smoking) and reflective motivation (e.g. foster a non-smoker identity). The selected intervention targets were judged likely to be best achieved by the intervention functions of education, persuasion, enablement, modelling, training and environmental restructuring [24], and 42 distinct BCTs from the BCTTv1 [54] were used to deliver the intervention content.

In accordance with the MOST development process [23, 58, 59], the next phase is for SmokeFree Baby to be evaluated in a factorial screening experiment to assess the effects of five intervention modules (identity change vs stress management vs health information vs promoting engagement with face-to-face support vs behavioural substitution) on the targeted behavioural outcomes (stopping smoking completely or cutting down). Pregnant smokers are randomly allocated to one of 32 experimental groups in a 2^5^ (2 × 2 × 2 × 2 × 2) full factorial design, in which each group receives a combination of the five modules and the different levels of each module (control vs full). Findings from this experiment will be used to inform intervention optimization by identifying components and component levels with the most potential to influence behaviour change. Depending on the findings, SmokeFree Baby will be revised and will either be tested in a second screening experiment or, if further evaluation is warranted, the app will be evaluated in a full-scale RCT.

Developing and optimizing digital smoking cessation interventions through iterative and multiphase processes are likely to take longer to complete than traditional RCTs. However, it is argued that this newer approach can advance intervention science faster by allowing researchers to systematically screen out ineffective intervention components and to keep only those components with the greatest potential to form better interventions [58]. The development of SmokeFree Baby shows that it is feasible to design an app to be evaluated in a factorial experiment to test a number of conditions simultaneously. This is because digital platforms permit high fidelity in delivering intervention components and a relatively straightforward random allocation of users to different experimental groups. Automatically collected data on pregnant smokers’ engagement with the app and helpfulness ratings on intervention components will be evaluated and used to inform decision-making in further iterations of the intervention development.

Limitations include the relatively limited extent to which pregnant smokers were actively involved in the early development of SmokeFree Baby in terms of what to include in the app, how to present the content and in what ways the intervention should be delivered to increase user engagement. However, a qualitative think-aloud study (reported separately) has since been conducted with pregnant smokers to explore their perspectives’ about the usability of the trial version of the app. As others have previously suggested [56], some decisions during the development of large and complex interventions, such as SmokeFree Baby, are likely to have been made without being documented. In order to mitigate this as much as possible, we kept detailed records of all stages of the intervention development process and reported a comprehensive description of these in the paper. Although SmokeFree Baby has been designed to be potentially useful for English-speaking pregnant smokers globally, the content was to a large extent designed to be relevant in the UK. It is also possible that the intervention will not be equally accessible across the social spectrum, as pregnant smokers from lower social grade groups might not have access to smartphones or have data allowance to download the app. Equity in access to digital support needs to be assessed, and data collected through SmokeFree Baby will provide insights into the socioeconomic characteristics of pregnant smokers who engage with the app.

To our knowledge, SmokeFree Baby is the first smoking cessation app that was specifically designed to meet the needs of pregnant smokers. The development was informed by a systematic application of theories, scientific evidence, BCTs and expert consensus in the research team. A rigorous methodology was followed from the early stages of intervention development that should facilitate multiphase intervention optimization in the future.

The findings reported in this manuscript have not been previously published, and the manuscript is not being simultaneously submitted elsewhere.

The data have not been previously reported.

The authors have full control of all primary data and agree to allow the journal to review the data if requested.

RW and IT are funded by a Centre grant from Cancer Research UK. JB’s post is funded by a fellowship from the UK Society for the Study of Addiction (SSA). The SSA and the National Centre for Smoking Cessation and Training provided funding for the app development.

## Electronic supplementary material

ESM 1 (DOCX 563 kb)
