# Supplementary material for: Development of SmokeFree Baby: a smoking cessation smartphone app for pregnant smokers
Source: Transl Behav Med. 2016 Oct 3;6(4):533–45. doi: 10.1007/s13142-016-0438-0 (PMC5110502; doi:10.1007/s13142-016-0438-0)
Supplement: Supplementary file 1 — (DOCX 563 kb) [file 13142_2016_438_MOESM1_ESM.docx]

Table S1: Principles generated from the COM-B model [[1](#_ENREF_1)] and PRIME theory [[2](#_ENREF_2)]

| I. Capability (psychological) |
| --- |
| 1. Improve knowledge of the health effects of smoking and benefits of smoking cessation. |
| 1. Improve knowledge of the types of smoking cessation support available. |
| 1. Improve skills to cope with cravings and stress. |
| 1. Improve self-regulatory capacity by adopting strategies that conserve mental energy. |
| II. Opportunity (social) |
| 1. Advise on and facilitate social support. |
| 1. Advise on avoiding social cues for smoking. |
| III. Opportunity (environmental) |
| 1. Provide easy access to stop smoking support resources in the locality or telephone helplines. |
| 1. Advise on avoiding environmental cues for smoking. |
| 1. Provide feedback on progress with stopping smoking (e.g. by including visual cues to show progress) |
| IV. Motivation (automatic) |
| 1. Provide distraction from urges to smoke to conserve mental energy. |
| 1. Provide support to cope with negative emotional states and stress. |
| 1. Provide positive reinforcements. |
| 1. Prompt pregnant smokers’ desire to stop smoking. |
| V. Motivation (reflective) |
| 1. Foster a non-smoker identity and associate it with positive mental images and feelings. |
| 1. Increase the salience of pregnant smokers’ identity as a ‘mother’/‘mum-to-be’ to promote cessation. |
| 1. Boost self-confidence in stopping smoking. |
| 1. Facilitate self-monitoring of smoking behaviour. |
| 1. Change beliefs regarding smoking and its effects to reduce stress. |
| 1. Advise on making a plan to stick to a personal rule: ‘not smoking at all no matter what’ or ‘cutting down to three or fewer cigarettes per day’. |

Table S2: Design principles

| I. Design principles adopted from the development of the StopAdvisor website [[3](#_ENREF_3)] |
| --- |
| 1. Use images (graphics or video) to convey information. |
| 1. Give control, choice and personal relevance. |
| 1. Keep text as brief as possible. |
| 1. Try to avoid grouping more than two sentences together. |
| 1. Navigation must be consistent and straightforward. |
| 1. Avoid a patronising tone in the text. |
| 1. Make the app as interactive as possible (e.g. questions, feedback, videos etc.) |
| 1. The app must look professional. |
| 1. Keep consistency throughout the app with regard to layout and grammar. |
| 1. Avoid small font size. |
| 1. Avoid replication. |
| 1. Remove all unnecessary words. |
| 1. Personalise as much as possible. |
| 1. Use ‘chatty’ everyday language, avoiding formality as much as possible. |
| 1. Express content in brief and specific terms. |
| 1. Feature an interactive component in each feature of the app (e.g. questions, text entry, videos etc.). |
| 1. Emphasize choice as much as possible. |
| 1. Make font consistent throughout the app. |
| 1. Keep number of fonts to a minimum. |
| 1. Remove unhelpful jargon and terminology. |
| 1. Manage expectations about the app in general and specifically about terms like personalised and tailoring by explaining them. |
| 1. Encourage regular use of the app to overcome the belief it should only be used if things are going badly. |
| 1. Personalise the source of SmokeFree Baby by expanding the ‘about the team’ section and adding smoking histories. |
| II. Design principles adopted from a study of optimal features of health-related websites [[4](#_ENREF_4)] |
| 1. Login procedures in the app have to be easy. |
| 1. Keep the length of the background questionnaire to the minimum. |
| 1. Present background questionnaire with a progress bar. |
| III. Design principles identified in a study of health care providers working with pregnant smokers [[5](#_ENREF_5)] |
| 1. Explain how the intervention works. |
| 1. Provide information in a format of ‘daily tips’. |
| IV. Design principles identified by the research team |
| 1. Include visuals and interactive elements in general app features and full modules. |
| 1. Use text only to convey information in the control version of each module. |
| 1. Make some content available pre-quit. |
| 1. Use teasers to content that can be unlocked at a later stage. |
| 1. Release new content throughout pregnancy. |
| 1. Reward daily logins with new content. |
| 1. Use daily push notifications to remind participants to log in the app. |
| 1. Use in-app notifications to signpost new content in various features. |

*Design principles that may promote user engagement.

Figure S1: Process of a multiphase intervention development of the SmokeFree Baby smartphone app


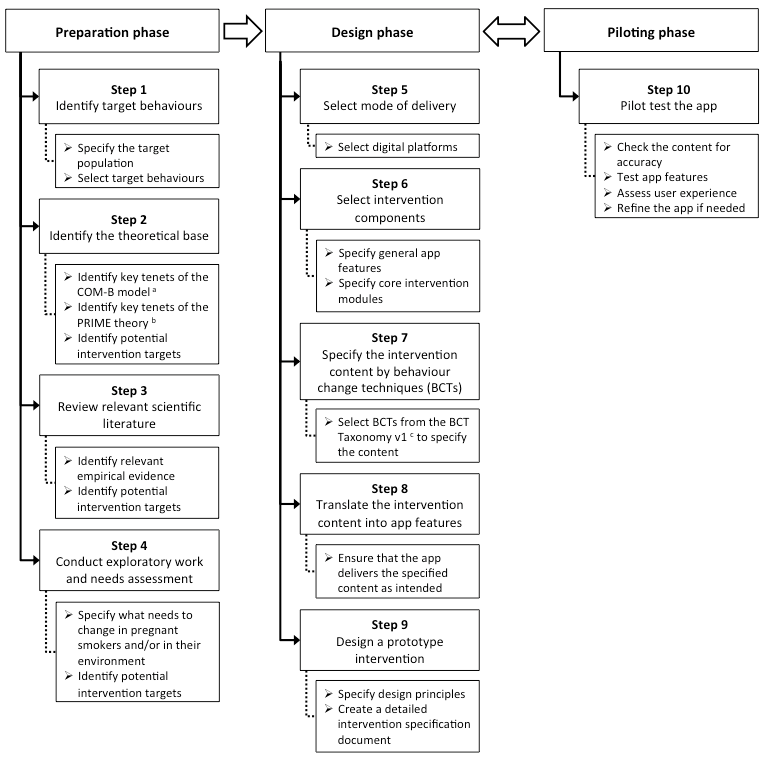


^a^ COM-B model [[1](#_ENREF_1)]; ^b^ PRIME theory [[2](#_ENREF_2)]; ^c^ BCTTv1 [[6](#_ENREF_6)]

Figure S2: Sample screenshots of the SmokeFree Baby app

**
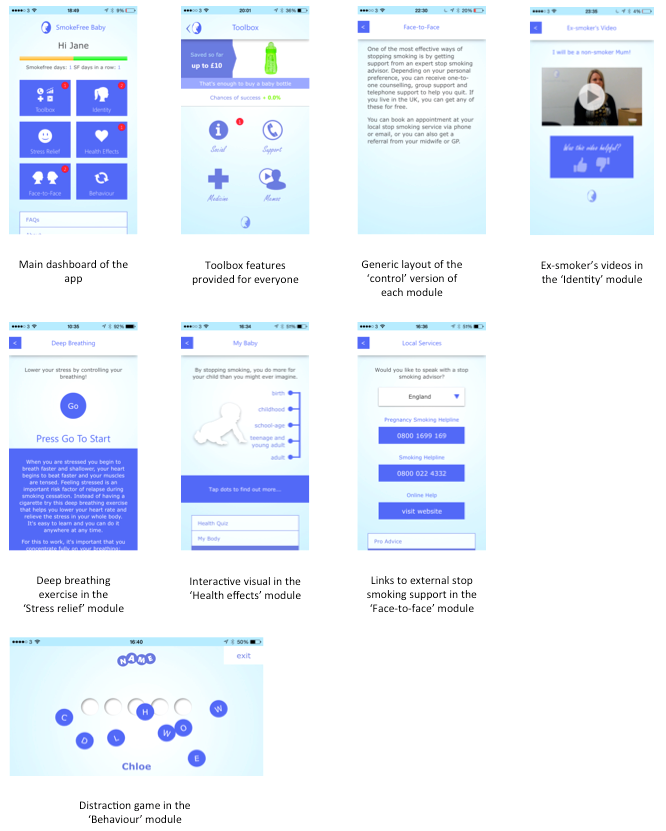
**

Figure S3: Structure of the core intervention modules

**
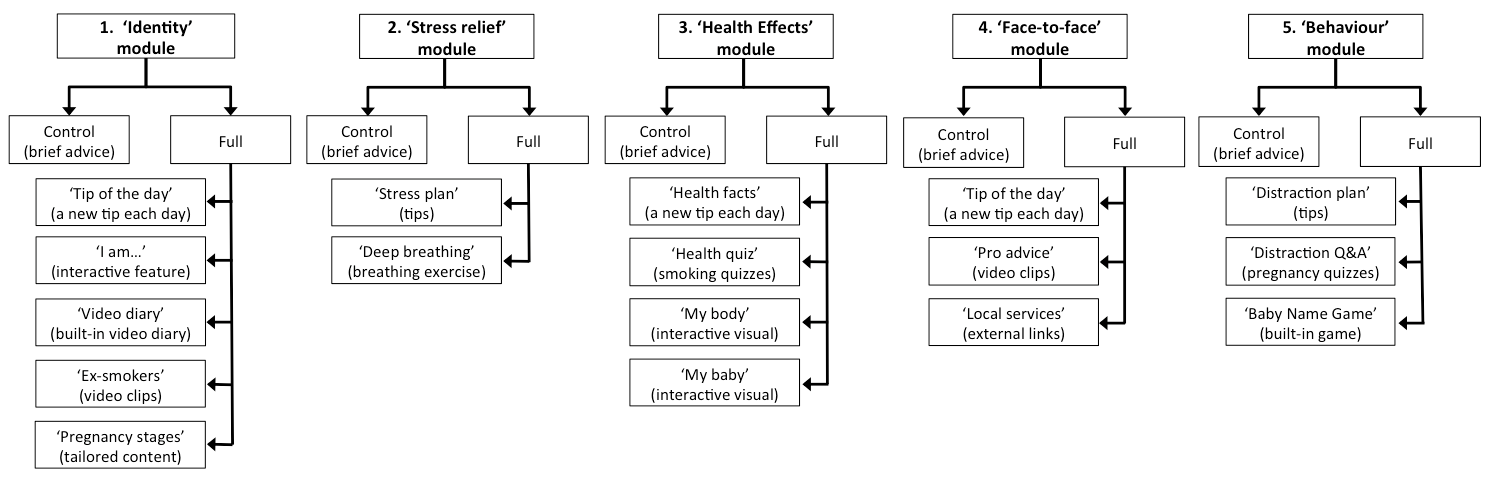
**

**References**

1. Michie, S., M.M. van Stralen, and R. West, *The Behaviour Change Wheel: a new method for characterising and designing behaviour change interventions.* Implementation Science, 2011. **6**(42).

2. West, R. and J. Brown, *Theory of addiction – Second Edition*. 2013, West Sussex, UK: Wiley Blackwell.

3. Michie, S., et al., *Development of StopAdvisor. A theory-based interactive internet-based smoking cessation intervention.* Translational Behavioral Medicine: Practice, Policy and Research, 2012. **2**(3): p. 263-275.

4. Schneider, F., L. van Osch, and H. de Vries, *Identifying factors for optimal development of health-related websites: a Delphi study among experts and potential future users.* JMIR Mhealth Uhealth, 2012. **14**(1): p. e18.

5. Tombor, I., et al., *Healthcare providers' views on digital smoking cessation interventions for pregnant women.* Journal of Smoking Cessation, 2015. **10**(2): p. 116-123.

6. Michie, S., et al., *The Behavior Change Technique Taxonomy (v1) of 93 hierarchically clustered techniques: building an international consensus for the reporting of behavior change interventions.* Annals of Behavioral Medicine, 2013. **46**(1): p. 81-95.
